# Supported Ionic Liquid Silica as Curing Agent for Epoxy Composites with Improved Mechanical and Thermal Properties

**DOI:** 10.3390/polym9100478

**Published:** 2017-09-29

**Authors:** Chongrui Zhang, Xiaoqian Mi, Junyu Tian, Junheng Zhang, Tiwen Xu

**Affiliations:** 1 Key Laboratory of Catalysis and Materials Science of the State Ethnic Affairs Commission & Ministry of Education, South-Central University for Nationalities, Wuhan 430074, China; 201421133047@mail.scuec.edu.cn (C.Z.); 201521132055@mail.scuec.edu.cn (X.M.); 201421133052@mail.scuec.edu.cn (J.T.); 2 School of Chemical Engineering, Guangdong University of Petrochemical Technology, Maoming 525000, China

**Keywords:** epoxy resin, silica, composites, supported ionic liquid, curing agent

## Abstract

The present study aims to improve the mechanical properties of epoxy composite by incorporating supported ionic liquid silica (IL-silica). The IL-silica not only showed improved interfacial interaction and reinforcement, but also served as cure agent of epoxy composites. The differential scanning calorimetry analysis revealed that epoxy composites could be successfully cured with IL-silica without any routine curing agents. IL-silica/epoxy composites presented higher mechanical and thermal properties compared with epoxy composite containing un-functionalized silica (u-silica). The dynamic mechanical analysis showed that the storage modulus of composites significantly increased with the addition of IL-silica in comparison to that with added u-silica, as well as the variation of *Tg* parameter. The incorporation of IL-silica simultaneously enhanced the tensile strength, toughness, and thermal stability of the epoxy composites. The considerable improvements in mechanical and thermal properties are ascribed to the improved dispersion of IL-silica and the enhanced interfacial interactions between epoxy matrix and IL-silica by strong covalent bonding, which results in an effective load transfer.

## 1. Introduction

At present, silica particles are widely employed as fillers to reinforce polymer composites. Silica particles can enhance the mechanical properties and overall performance of polymer composites [1,2]. However, silica particles tend to agglomerate in polymer matrix, the high polarity of the silica particles’ surface with silanol groups (Si–OH) and weak adhesion between silica and polymer matrix have restricted the reinforcement effect. Researchers have put forth a great deal of effort to make silica fillers disperse uniformly in polymer matrix and enhance the interfacial property between silica and polymer [3,4]. A wide variety of chemical and physical treatments are applied to modify silica particles for improving the dispersion and interface properties [5,6,7]. Guo utilized a phosphonium ionic liquid (PIL) as a novel catalyst for the silanization reaction between silica and bis(3-triethoxysilylpropyl)-tetrasulfide (TESPT) in silica-filled rubber composites, and the resulting composite exhibited a superior overall performance [8]. Recently, we used epoxidized natural rubber (ENR) as an interfacial modifier to improve the compatibility of silica in a polymeric matrix, and thus produced an increase in the mechanical properties of natural rubber/silica composites [9].

Epoxy resins have evolved as one of the most important thermosetting resins because of their good creep resistance, high strength, good thermal stability, and structural stiffness [10,11]. However, there are some critical properties that hindered the applications of epoxy resin; for example, they are relatively brittle and have low fracture toughness, which need to be improved in the application of advanced composites. Numerous methods have been designed with the goal of increasing the toughness of epoxy resin [12,13]. The most common technique over the past decades was the addition of elastic phase in epoxy resin such as carboxyl-terminated butadiene-coacrylonitrile (CTBN) or poly(ether sulfone) thermoplastic polymers [14,15]. This technique can drastically enhance the toughness of epoxy resin but also significantly reduce its modulus and thermal properties. To overcome the limitations of elastomers and thermoplastic polymers, nanoscale reinforcements have been developed in the past decades [16,17]. Recently, Hadavinia summarized the effects of nanoparticle types, dispersion, and interfacial strength on the Young’s modulus (E), ultimate tensile strength (UTS), and fracture toughness of epoxy composites [18]. The addition of nanofillers—especially silica particles—has already provided significant reinforcing effects on the mechanical properties of epoxy resin, especially impact resistance and fracture toughness [19,20]. However, the dispersion of silica is still a challenge for epoxy composites with high fracture toughness [21,22]. The formation of agglomerates would result in a reduction of the toughness, specifically when the volume fraction is high. Another challenge in employing silica is the weak interfacial bonding between silica and polymer matrix, resulting in the deleterious effect of silica-reinforced composite [23,24].

**Figure 1:**
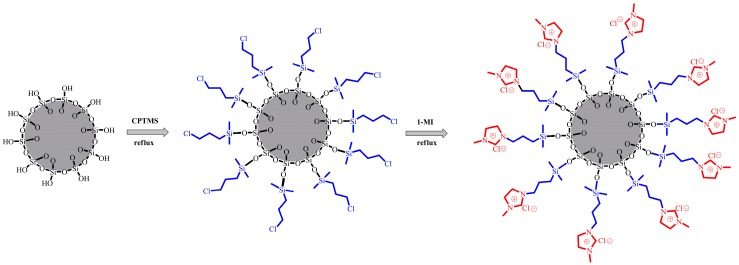
Scheme of supported ionic liquid silica (IL-silica). CPTMS: 3-chloropropyltrimethoxysilane; 1-MI: 1-methylimidazole.

This work focuses on supported ionic liquid silica (IL-silica), in an attempt to achieve uniform dispersion and strengthened interfacial bonding of epoxy composites. The surface of silica was modified with 3-chloropropyltrimethoxysilane and 1-methylimidazole (see Figure 1). The effects of IL-silica on cure behavior, mechanical and thermal properties of epoxy composites with different filler weight fractions were demonstrated. The results of the measured mechanical and thermal properties of epoxy composites were compared with an un-functionalized silica (u-silica) system. The results show that the IL-silica can be used as curing agent for epoxy composites. The integration of IL-silica in epoxy composites resulted in the simultaneous improvement of mechanical and thermal properties of composites. Microscopy of the fracture surfaces was conducted using scanning electron microscopy (SEM) to identify the failure mechanisms of epoxy composites.

## 2. Materials and Methods 

### 2.1. Materials

Silica (specific area of 210 m^2^) was obtained from Wanzai County Huiming Chemical Industry Co., Ltd., Yichun, China. 3-Chloropropyltrimethoxysilane (CPTMS), 1-methylimidazole (1-MI), and xylene were supplied by Aladdin Industrial Corporation (Shanghai, China). Diglycidyl ether of bisphenol A (DGEBA) (epoxy equivalent weight = 190–195 g/eq) was purchased from Yueyang Resin Factory, Yueyang, China. Alkyl (C12-C14) glycidyl ether (AGE) (epoxy equivalent weight = 275–300 g/eq) was supplied by Hexion Inc., Shanghai, China.

### 2.2. Preparation of Supported Ionic Liquid Silica (IL-Silica)

The synthetic route of IL-silica was as follows: 1.00 g silica was added in 100 mL xylene and the suspension was sonicated for 30 min. The obtained supernatant and 0.29 g 1-methylimidazole, 0.70 g 3-chloropropyltrimethoxysilane were loaded into a 250 mL three-necked flask with stirring at 80 °C for 12 h under reflux condition in nitrogen atmosphere. The slurry mixture was filtered and washed with acetone to eliminate any un-reacted chemicals. Finally, the filtercake was dried under vacuum at 60 °C for 24 h.

### 2.3. Composite Fabrication

In order to get well dispersed fillers in epoxy matrix, the epoxy composites were prepared by the following procedures. An S-65 three-roll mill (Chang Zhou Wu Jin Ba Fang Mechanic Factory, Changzhou, China) was used to mix fillers with epoxy resin. The IL-silica was mechanically mixed with DGEBA and AGE on a hotplate and then transferred on the three-roll mill. The gap width between the rolls was set to 2 μm and the velocities of the three rolls were set to 32/89/250 rpm. Three processing cycles were performed. After each cycle, the mixture was refilled into the feed opening and the rollers were cleaned. The mixture was finally transferred to a silicone rubber mold and cured at 80 °C for 2 h and 120 °C for 2 h, followed by a post-curing at 150 °C for 2 h.

The detailed components of epoxy composites are shown (in parts per hundred epoxy, phr) in Table 1. In addition, the 1-MI was fixed at 1.62 phr in the control sample. The amount of bonded 1-IM on the surface of the silica was determined by the residue weight of IL-silica after being heated to 600 °C and calculated to ensure the equivalent 1-MI component was present in epoxy composites. AGE was used as reactive diluent and composited with DGEBA to decrease viscosity.

### 2.4. Characterizations

Fourier transform infrared (FT-IR) spectroscopy was measured on a Nicolet NEXUS470 spectrometer (Nicolet, Madsion, WI, USA). Themogravimetric analysis was carried out on Netzsch TG209 F3 instrument (NETZSCH-Gerätebau GmbH, Selb, Germany) with a heating rate of 10 °C/min from 30 to 600 °C. X-ray photoelectron spectroscopy (XPS) was performed on an X-ray photoelectron spectrometer VG Multilab 2000 (Thermo Electron Corporation, Waltham, MA, USA) with an aluminum Kα source (1486.6 eV). Evaluation of cure kinetic parameters was performed by differential scanning calorimetric analysis (NETZSCH-Gerätebau GmbH, Selb, Germany). About 5–10 mg of sample was heated from room temperature to 250 °C in N_2_ atmosphere. Tensile tests were carried out on an AI-700M universal test machine (Gotech Testing Machines Inc., Dongguan, China) according to ASTM D638 (2014) at a crosshead speed of 1 mm/min. Flexural tests were performed on an AI-700M universal test machine in accordance with ASTM D790 (2017) with a speed of 1 mm·min^−1^. The un-notched Izod impact strength was measured on a GT-7045-MDL pendulum impact test machine (Gotech Testing Machines Inc., Dongguan, China) according to ASTM D4812 (2011). Dynamic mechanical analysis (DMA) was analyzed using a DMA Q800 (TA instruments, Newcastle, DE, USA) in single cantilever mode with a heating rate of 5 °C/min and a frequency of 1 Hz over a temperature range from room temperature to 200 °C. Scanning electronic microscope (SEM) was carried out with a SU8010 (Hitachi, Tokyo, Japan).

## 3. Results

**Figure 2:**
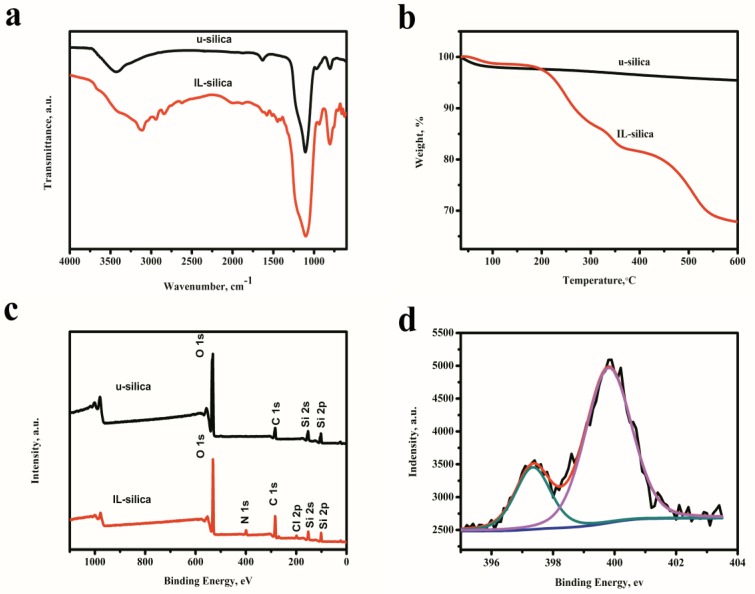
Characterization of (**a**) Fourier transform infrared (FT-IR) spectroscopy; (**b**) Thermogravimetric analysis (TGA) curves; (**c**) X-ray photoelectron spectroscopy (XPS) broad scan spectra; and (**d**) XPS N 1s curves of silica and IL-silica.

Figure 2a shows the FT-IR spectra of u-silica and IL-silica. For u-silica, the bonds at 3430 and 1630 cm^−1^ correspond to the absorption of silanol groups and hydroxyl groups of the adsorbed water. The stretching vibration of Si–OH group is located at 961 cm^−1^. The peaks at 1110 and 804 cm^−1^ are assigned to the stretching vibrations of Si-O-Si group. In the FT-IR spectra of IL-silica, the bands at 2940 and 2839 cm^−1^ are due to the symmetric stretching band νs(C–H) and asymmetric stretching band νas(C–H) of alkyl groups. The imidazole ring vibrations are observed at 1575 and 1510 cm^−1^. The peak at 1446 cm^−1^ is assigned to the combined band of imidazole ring vibration and deformation band δ(C–H).

The thermogravimetric analysis (TGA) curves of u-silica and IL-silica are shown in Figure 2b. The major weight loss of u-silica between 30 and 200 °C is ascribed to the residual water. For IL-silica, the first region is only about 2.5% weight loss in the range of 30–200 °C. The second region in the range of 200–600 °C is due to the thermal cracking of propyl and imidazole segments. The 1-methylimidazole weight percentage calculated from the TGA curves was 8.1%, corresponding to 0.99 mmol 1-methylimidazole per 1 g of IL-silica.

The XP Survey and N1s high resolution XPS spectra of u-silica and IL-silica are presented in Figure 2c,d. In the spectra of IL-silica, the peaks at 198.6 and 399.9 eV correspond to Cl 2p and N 1s, respectively. The spectrum of N1s splits into two peaks, which justifies the two nitrogen atoms in the structure of the supported imidazole ionic liquid.

**Figure 3:**
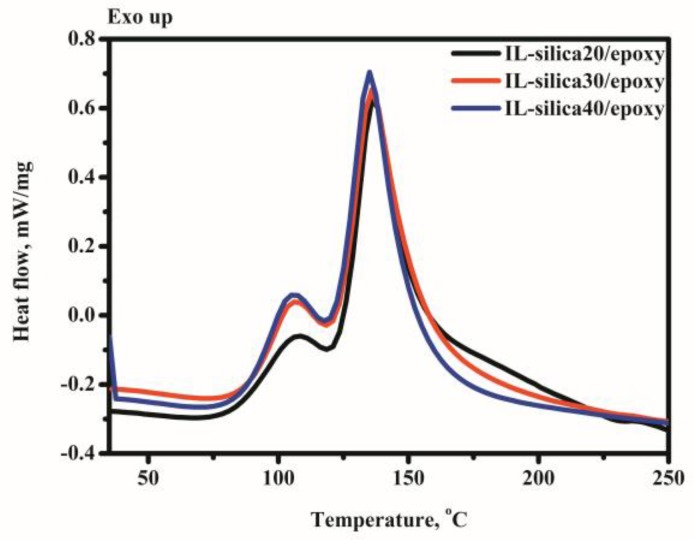
Differential scanning calorimetry (DSC) curves of epoxy composites with 20, 30, and 40 phr IL-silica heated at 5 °C/min.

**Figure 4:**
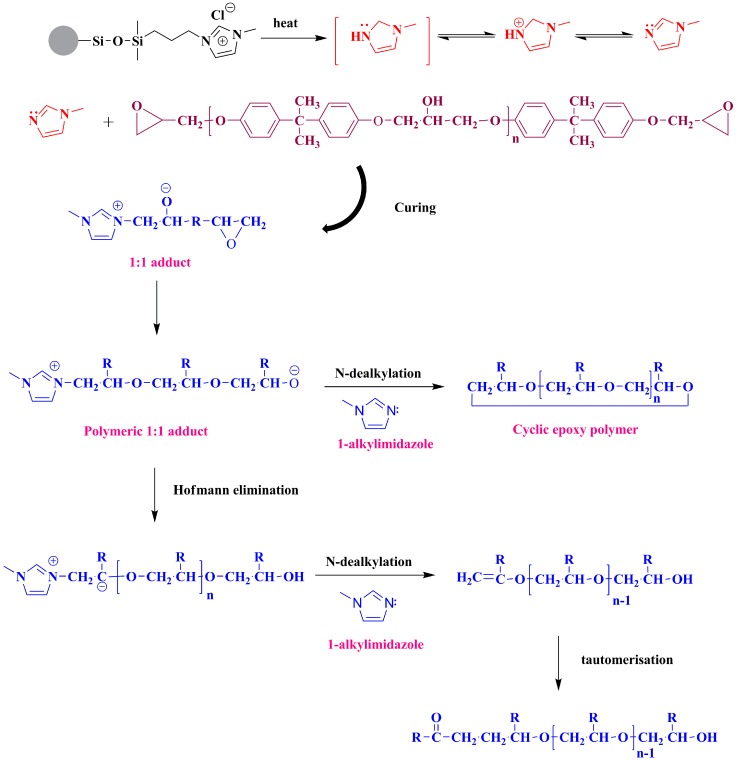
Schematic of cross-linking reactions between epoxy resin and IL-silica.

Figure 3 is the dynamic differential scanning calorimetry (DSC) thermographs of IL-silica and DGEBA mixtures at IL-silica concentrations between 20 and 40 phr. It is observed that there is a dual reaction exotherm for all systems. The onset curing temperatures of the first exothermic peak are around 105–108 °C, the second exotherms are 135–137 °C. For an increase in the IL-silica concentration from 20 to 40 phr, the heat of reaction (ΔH) decreases from 450.8 to 406.3 J. The possible reaction mechanisms of epoxy cured with imidazolium-based ionic liquids have been presented by Palmese and Spychaj [25,26,27,28]. In the present work, the proposed cure reaction of DGEBA crosslinked with IL-silica is shown in Figure 4. At relatively lower temperature, the thermal decomposition of supported imidazole ionic liquid occurs so as to form a relatively stabilized *N*-heterocyclic carbon structure. In the initiation step of curing, the pyridine-type nitrogen atom of imidazole ionic liquids reacts with epoxy groups to form 1:1 adducts. In the following process, the pyrrole-type nitrogen of 1:1 adducts transforms to pyrridine-type nitrogen with H^+^ transfer. The formation of the above pyridine-type nitrogen can further react with epoxy polymer chains by combination of 1:1 adducts via anionic polymerization. Furthermore, two possible options would form in the cross-linked networks, both of which lead to the regeneration of 1-alkylimidazoles: (i) the cyclic epoxy polymer chains are formed through *N*-dealkylation reaction; (ii) the unsaturated structures are formed from the epoxy cross-link structure with carbonyl groups through tautomerization.

**Figure 5:**
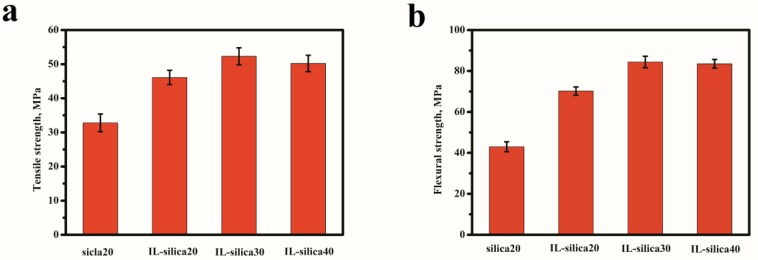

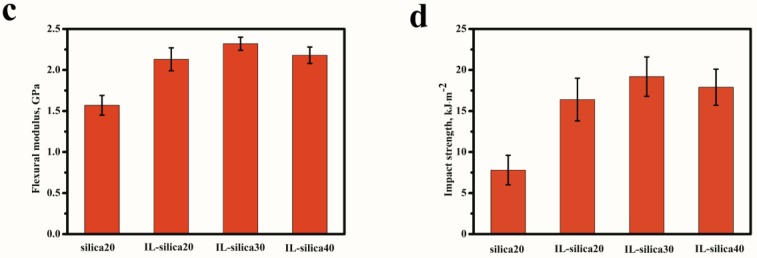
Mechanical properties of epoxy composites containing u-silica and IL-silica. (**a**) Tensile strength; (**b**) Flexural strength; (**c**) Flexural modulus; (**d**) Impact strength.

Figure 5 exhibits the mechanical properties of epoxy composites with different IL-silica loading. Compared with u-silica20/epoxy composites, the tensile strength, flexural strength, flexural modulus, and impact strength of IL-silica20/epoxy composites increased from 32.8 MPa, 43.0 MPa, 1.57 GPa, and 7.8 kJ·m^−2^ to 46.1 MPa, 70.2 MPa, 2.13 GPa, and 16.4 kJ·m^−2^. The improvement is due to the uniform dispersion of fillers and chemical bonds at the matrix/filler interface via polymerization of epoxy resin with the active imidazole groups of IL-silica. The reinforcement effect of polymer composites largely depends on the efficiency of load transfer at the interface [29,30]. In the present epoxy network, IL-silica served as a linker for epoxy polymer chain. This can efficiently improve the interactions between epoxy matrix and the silica filler. The uniform dispersion of IL-silica and improved interfacial interaction are favorable to enhance the load transfer from epoxy matrix to silica filler, and thus significantly increase the mechanical properties [31,32]. By using 30 phr IL-silica in epoxy composite, tensile strength increased from 46.1 to 52.3 MPa, flexural strength from 70.2 to 84.4 MPa, flexural modulus from 2.13 to 2.32 GPa, and impact strength from 16.4 to 19.2 kJ·m^−2^. A further increase in IL-silica content (up to 40 phr) generated a slight decrease of mechanical properties. This can be explained by the agglomeration of IL-silica in high content.

**Figure 6:**
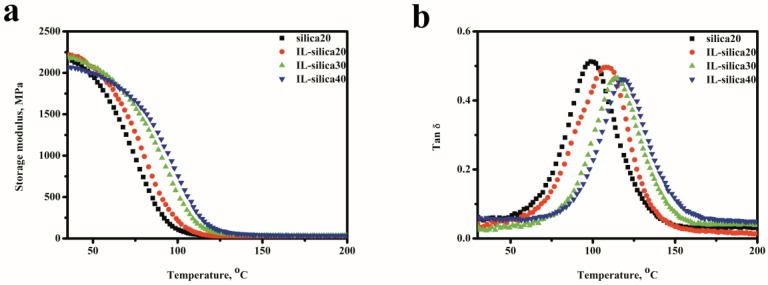
Dynamic mechanical properties of epoxy composites containing u-silica and IL-silica. (**a**) Storage modulus; (**b**) tan δ.

Here the thermomechanical properties of epoxy composites are investigated based on the DMA results. The storage modulus and tan δ of various epoxy composites are shown in Figure 6. The homogeneous dispersion of fillers in polymer matrix and their interfacial interaction will have a significant effect on the thermomechanical properties [33,34]. For epoxy composites containing IL-silica, IL-silica can be used both as curing agent and effective reinforce filler for epoxy composites. Epoxy composite with IL-silica exhibited higher storage modulus in the rubbery region than the composite filled with u-silica. The storage modulus E′ of the epoxy composites in the rubbery region increased by increasing the IL-silica content. This behavior is attributed to the improved interfacial interactions, since the IL-silica can discourage the mobility of epoxy matrix around IL-silica and provide additional stress transfer under loading. In IL-silica/epoxy composites, imidazole groups of IL-silica could react with epoxies and construct the crosslink network through covalent bonds on the interface. This effectively restricts the movement of epoxy polymer chains and promotes energy dissipation from the matrix to filler, resulting in an increase in modulus. Figure 6b shows the temperature dependence of tan δ values of epoxy composites. Clearly, the *Tg* value of the IL-silica20 sample shifted toward higher temperature by 9.2 °C compared to that of the silica20 sample. The presence of IL-silica led to a higher cross-linking degree of the resultant network than u-silica/epoxy composites. The *Tg* value of IL-silica/epoxy composites continuously shifted to higher temperature with increasing IL-silica content. This can be attributed to the increase of crosslinks density of epoxy resin with increasing IL-silica content, which would restrict the mobility of the epoxy chains for the enhanced interfacial interactions.

**Figure 7:**
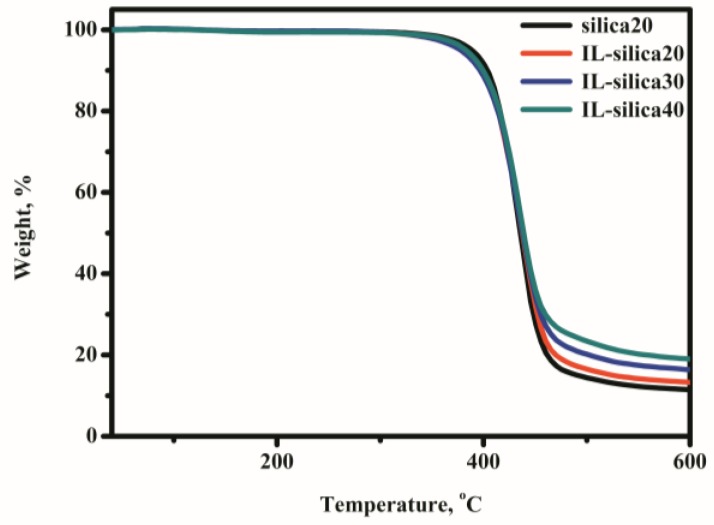
TGA thermograms of epoxy composites containing u-silica and IL-silica.

The TGA curves of epoxy composites are presented in Figure 7. The presence of IL-silica enhanced the thermal stability of epoxy composites, as both the decomposition temperature (*T*_onset_) and *T*_10%_ shifted toward higher temperatures than that of the composite containing u-silica. For the composites containing various loadings of IL-silica, the *T*_onset_ and *T*_10%_ increased with increasing IL-silica content. This can be explained in terms of the improved interfacial interaction and increased cross-linking density of epoxy composites [35,36,37].

**Figure 8:**
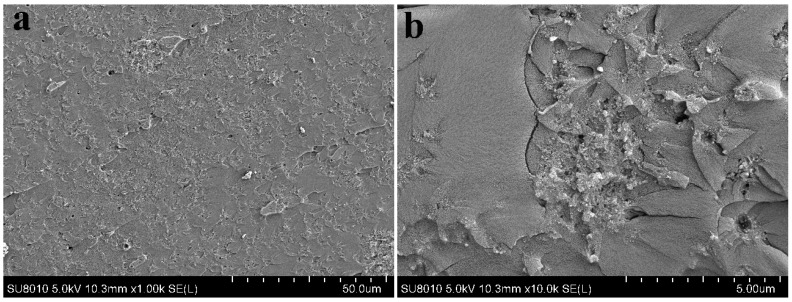

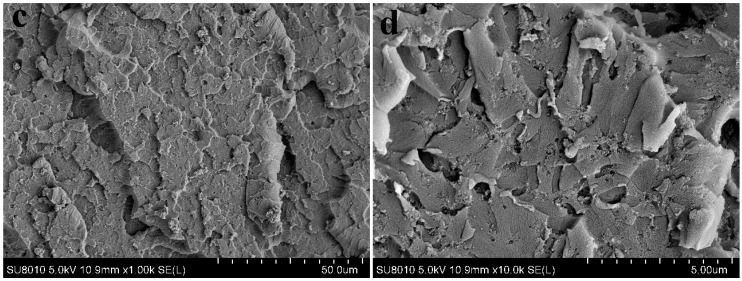
SEM morphologies of impact fractures of epoxy composites: (**a**,**b**) Epoxy composites containing u-silica (silica20); (**c**,**d**) Epoxy composites containing IL-silica (IL-silica20).

Figure 8 presents the representative morphologies of the fractured surfaces of epoxy composites. From Figure 8a,b, the u-silica is present in the form of agglomerates in the epoxy matrix due to the formation of hydrogen bonds among abundant silanol groups, which partially offsets the reinforcement effect on mechanical properties. In case of IL-silica/epoxy composites, most of the silanol groups have been reacted and the surface has been changed from hydrophilic to hydrophobic, thus the agglomeration tendency would be reduced. As shown in Figure 8c,d, the absence of obvious aggregates on the fractured surface confirms the more uniform dispersion of IL-silica in epoxy resin than u-silica. Clearly, the IL-silica is embedded well into and tightly bound to the epoxy matrix. Additionally, in Figure 8c,d, large and elongated crack patterns confirm higher crack growth resistance of IL-silica/epoxy composite, which facilitates energy dissipation and increases the fracture toughness [38]. These characteristics are consistently associated with the increased interfacial adhesion and efficient transfer of the load in IL-silica/epoxy composites.

## 4. Conclusions

This study demonstrated supported ionic liquid silica (IL-silica) as curing agent for epoxy composite with improved dispersion and interfacial interaction. Epoxy composites were successfully cured with IL-silica without the addition of any routine curing agent. The IL-silica were observed to be highly dispersed and well integrated in epoxy resin compared to un-functionalized silica (u-silica). The uniform dispersion and improved interfacial adhesion provide for an efficient load transfer from the epoxy matrix to silica and thus enhance the overall mechanical and thermal properties of epoxy composites.

## Figures and Tables

**Table 1:** The formulation of epoxy composites. DGEBA: diglycidyl ether of bisphenol A; IL-silica: ionic liquid silica; u-silica: un-functionalized silica.

Sample	DGEBA	AGE	u-Silica	IL-Silica	1-MI	CPTMS
silica20	100	15	14.46	0	1.62	3.92
IL-silica20	100	15	0	20	0	0
IL-silica30	100	15	0	30	0	0
IL-silica40	100	15	0	40	0	0
